# Correlation of Computed Tomography, Pathological Findings, and Clinical Outcomes for Appendicoliths in Appendicitis

**DOI:** 10.1097/AS9.0000000000000280

**Published:** 2023-04-18

**Authors:** Zachary N. Weitzner, Alex Chung, Bita V. Naini, Danielle Graham, Edward H. Livingston

**Affiliations:** From the * Department of Surgery, David Geffen UCLA School of Medicine, Los Angeles, CA; † Department of Radiology, David Geffen UCLA School of Medicine, Los Angeles, CA; ‡ Department of Pathology and Laboratory Medicine, David Geffen UCLA School of Medicine, Los Angeles, CA.

**Keywords:** Appendicitis, appendicolith, obstruction, fecalith

## Abstract

**Objectives:**

To correlate preoperative imaging of fecaliths with what is seen in surgical specimens.

**Background:**

Early studies considered radiological findings of appendicoliths as a contraindication for nonoperative treatment of appendicitis. There is no standard definition for what is labeled as an appendicolith radiologically and little is known about the pathological correlates of these lesions.

**Methods:**

A single center, retrospective study of a consecutive series of adult patients who underwent appendectomy for acute appendicitis from March 2021 to February 2022 was performed. The primary outcome was concordance between preoperative cross-sectional imaging description of appendicolith with postoperative gross pathology description. Images were retrospectively reviewed by an independent radiologist, and the presence and characteristics of appendicoliths and appendices were examined.

**Results:**

Of 88 cases of appendicitis, 86 were diagnosed preoperatively by computed tomography (CT) imaging. Appendicoliths were seen either on CT or pathology in 45 (51%) patients. Of these 45 patients, a total of 38 (84%) were identified radiographically, and 28 (62%) were identified on pathology. Of the 38 appendicoliths diagnosed on preoperative imaging, only 21 (55%) were confirmed pathologically. Additionally, of the 28 appendicoliths observed on pathology, only 21 (75%) were identified preoperatively on imaging. There was no appendiceal obstruction in 10 of the 40 cases (25%) in which retrospective radiological review identified appendicoliths.

**Conclusions:**

Discrepancies were observed between CT and pathology findings of appendicoliths. Not all appendicoliths seem to cause appendicitis. Because the presence of appendicolith influences the treatment decisions, there is a need to standardize their radiological diagnosis and better understand their pathophysiology.

## INTRODUCTION

Appendicoliths, or fecaliths, are accumulations of fecal material lodged in the appendix. Originally described by Reginald Fitz when he characterized appendicitis in 1886, the clinical significance of these lesions is unclear.^1^ The pathological underpinnings of appendicitis are not known but obstruction of the appendiceal lumen by appendicoliths makes for an attractive explanation. Nevertheless, even when Fitz first described appendicoliths, he noted that they were frequently found in the lumen of normal appendices, demonstrating that the presence of appendicoliths does not always coincide with appendicitis.

Until recently, appendicoliths were more of a curiosity than a pathologically relevant finding. In one of the first randomized controlled trials examining the treatment of appendicitis with antibiotics alone, the presence of appendicoliths was noted to possibly correlate with higher failure rates.^2^ This observation led the next set of investigators examining nonoperative treatment of appendicitis (NOTA) to exclude patients who had appendicoliths from their study altogether.^3^ A subsequent pragmatic randomized controlled trial investigating NOTA did include patients who had radiologic evidence of appendicoliths, but found an approximately 10% higher failure rate of nonoperative treatment.^4^

As NOTA becomes more common, appendicoliths have increased in importance as their presence may influence the treatment decisions for both patients and providers. Despite their newfound significance in clinical decision-making, there is no precise definition of what is deemed an appendicolith. The purpose of this study was to correlate the computed tomography (CT) and the pathology findings of appendicoliths so that the prognostic significance of these lesions can be better understood.

## MATERIALS AND METHODS

### Study Population and Data Source

We retrospectively reviewed a consecutive series of adult patients older than 18 years who underwent appendectomy at a single-academic quaternary care center between March 1, 2021 and March 1, 2022. Patients were identified by review of all operating room logs, finding instances where an appendectomy was performed. Patients treated nonoperatively for appendicitis were not considered for inclusion in this study. Patients diagnosed as having appendicitis without imaging were excluded as were patients undergoing oncologic resection for appendiceal neoplasms.

The medical record was reviewed to obtain demographic data, clinical information, imaging results, and pathology reports. Data were collected and stored in a secure, Health Insurance Portability and Accountability Act compliant web-based database. All specimens were reviewed in detail by UCLA’s Department of Pathology using a protocol specifying how the appendiceal specimens should be processed. Specifically, appendectomy specimens were opened to examine for the presence of appendicoliths, and the presence or absence of them was noted. If appendicoliths were seen grossly, their size, number, presence of calcification, and whether they appeared to be free-floating or obstructing were recorded.

### Outcomes

The primary outcome was concordance between preoperative cross-sectional imaging description of appendicolith and postoperative gross pathology description. After the concordance was assessed between preoperative imaging and pathological findings, the CT scans were re-reviewed for all the patients by an independent radiologist (A.C.) not involved in reading the preoperative images used for clinical decision-making. The radiologist performed a focused examination of the appendix on the CT examinations, identified appendicoliths and other relevant CT findings, measured the appendices dimensions and appendicolith Hounsfield units (HU). To increase radiographic sensitivity and minimize false-negative results with respect to pathology standard, radiographic appendicolith was defined as a discreet focus of hyperdensity measured in maximum HU of at least 20 HU greater than appendiceal wall and lumen maximum HU.

### Statistical Analysis

Descriptive data were analyzed for all study patients. We compared patient characteristics for those who had or did not have radiographic and pathologic concordance of appendicolith findings. Summary data were presented as the number of patients (n) with percentage (%) and either median with interquartile range for nonnormally distributed data or mean with SD for normally distributed data. Statistical significance for group differences was determined by Student *t* test for continuous data and either Pearson χ^2^ or Fisher exact tests for categorical variables. Receiver operator curves (ROC) were calculated and plotted for the true positive rate versus false positive rate (1-sensitivity) using the R program rROC. True positives were defined as having a CT finding of an appendicolith as seen upon re-review of the CTs by A.C., confirmed by an appendicolith observed at the time of pathological examination. False positives were defined as no appendicolith found at pathology when one was reported on the preoperative CT. Likelihood ratios (LR) were calculated based on the resultant sensitivity and specificity.^5,6^ Any *P*-values ≤0.05 were considered statistically significant.

### Ethical Approval

This study design was independently reviewed by the University of California, Los Angeles Institutional Review Board before initiation and was deemed Institutional Review Board exempt.

## RESULTS

**FIGURE 1.**
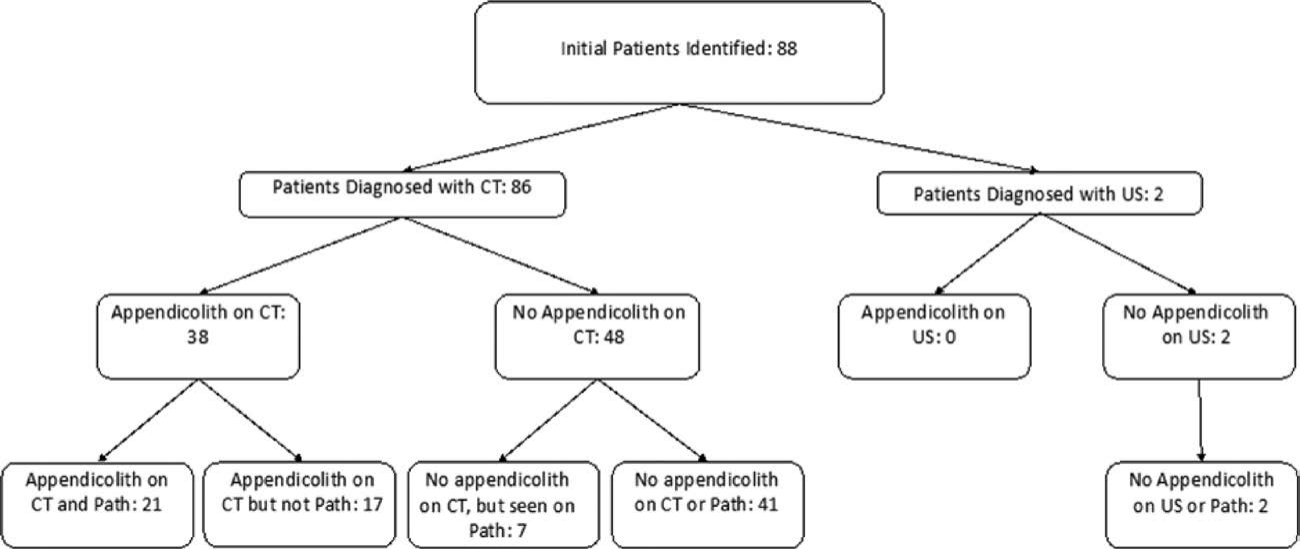
CONSORT diagram for flow of patients through the study.

Between March 1, 2021 and March 1, 2022, a total of 88 patients were identified who underwent appendectomy (Fig. 1). Of these, 86 were initially diagnosed with appendicitis by CT and 2 were diagnosed with ultrasound. None underwent operations based on clinical diagnosis alone. Because we were comparing CT and pathological findings, the 2 patients who did not have CT imaging were excluded from the data analyses. Both of these patients were female, one of whom was pregnant, and neither had appendicoliths observed on pathology or ultrasound.

Demographic data of the included cohort is displayed in Table 1. Mean age was 37.3 years and patients were evenly distributed between male and females. Most patients identified were White, and 36% identified as Hispanic. No patients diagnosed with CT were pregnant. There was 1 case of stump appendicitis and 1 case of interval appendectomy included in the cohort.

**TABLE 1.** Demographics of the Patient Population

N = 86	
Age [mean, (SD)]	37.3 (17.8)
Male [n (%)]	43 (50%)
Race [n (%)]	
Asian	12 (14.0%)
Black	3 (3.5%)
White	71 (82.6%)
Ethnicity [n (%)]	
Hispanic	31 (36.0%)
Non-Hispanic	55 (64.0%)
Pregnant [n (%)]	0 (0%)
Interval appendectomy	1 (1.2%)
Stump appendicitis	1 (1.2%)

The severity of illness is shown in Table 2. Overall, patients were relatively healthy, with no patients presenting with qSOFA scores >1 (range 0–3 with 3 representing a high risk for severe consequences of sepsis).^7^ Tachycardia was relatively common, present in 23% of patients, and hypotension (systolic blood pressure ≤100 mm Hg) was observed in 13% of patients. Fever (temperature >38.0°C) was relatively uncommon, seen only in 8% of patients, and only 1 patient presented with altered mental status, although this was later determined not to be a result of infectious causes.

**TABLE 2.** Illness Severity

n (%)	
Febrile (T > 38.0 C)	7 (8.1%)
Tachycardia (HR >100)	20 (23.3%)
Altered mental status	1 (1.2%)
Hypotension (SBP ≤ 100)	11 (12.8%)
qSOFA score	
0	70 (81.4%)
1	16 (18.6%)
2	0 (0%)
3	0 (0%)

HR, Heart Rate; SBP, Systolic Blood Pressure; qSOFA, quick Sequential Organ Failure Assessment.

Appendicoliths were identified in either CT or pathological examination in 48 of the 86 (52.3%) cases of appendicitis (Table 3). Appendicoliths were identified in 38 (44%) CTs and 28 (33%) of pathological examinations. Only 21 (24%) appendicitis cases had appendicoliths observed in both the CT and pathological examinations. If a pathology finding of an appendicolith is considered the reference standard for the presence of these lesions, the sensitivity (true positive/all positive tests) of CT for finding an appendicolith when it is observed on CT was 21 of 28 (75%). The specificity (true negative/all negative tests) for appendicolith actually being absent when not seen on CT was 41 of 58 (70.7%).

**TABLE 3.** Correlation of CT and Pathology for Appendicoliths

	Appendicolith on Pathology	No Appendicolith on Pathology	Total
Appendicolith on CT	21	17	38
No appendicolith on CT	7	41	48
Total	28	58	

**FIGURE 2.**
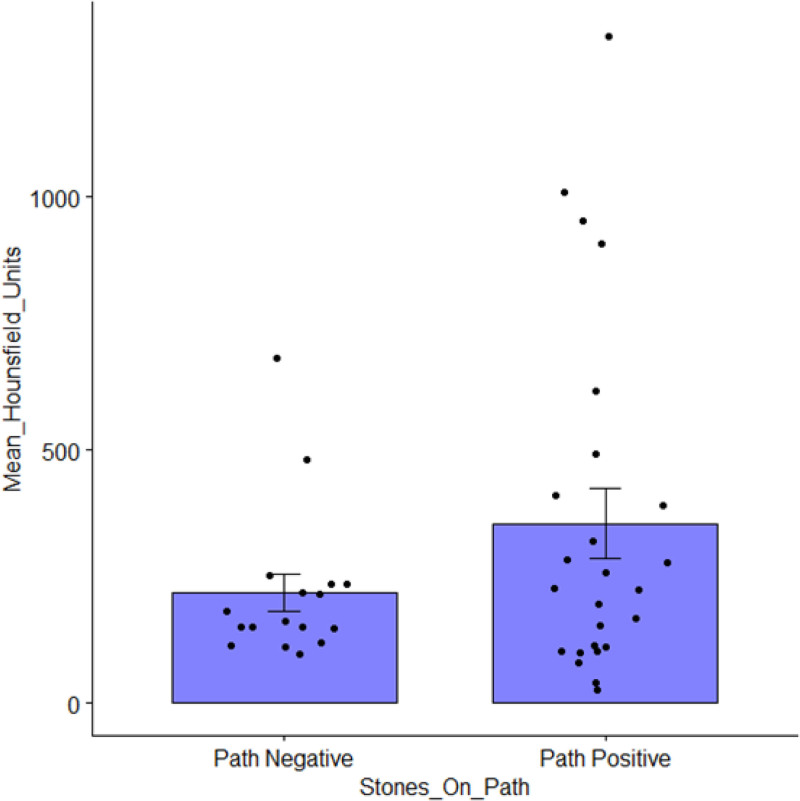
Distribution of Hounsfield units of appendicoliths observed on CT before appendectomy stratified by appendicolith observation during pathological examination of the specimen. The mean ± SEM Hounsfield units for appendicoliths visualized on CT but not seen on pathology was 217 ± 36 and for those seen on pathology it was 355 ± 69, *P* = 0.13 (*t* test).

An independent radiologist (A.C.) retrospectively reviewed the 46 cases in which an appendicolith was identified either on CT or by pathology (Table 4). Of the 7 instances where appendicoliths were found at pathology but not reported on the preoperative CT scan, retrospective review did note radiographic evidence for appendicoliths in 4 of the 7 cases. Detailed review of these cases demonstrated high frequencies of periappendiceal inflammation and appendiceal dilation (89.1% and 87.0%, respectively). The median (interquartile range) radiodensity of the appendicoliths was 206 HU (114–301) with the median maximal radiodensity of the appendicoliths being 264 HU (182–453). Radiodensity of appendicoliths was highly variable, with some appendicoliths having foci of radiodensity upwards of 1300 HU, whereas other appendicoliths had maximal radiodensities as low as 116 HU. Figure 2 plots the HU of appendicoliths stratified by their being or not being observed when examined pathologically. Most of the HU measurements are scattered between 100 and 300 with only a relatively small number of appendicoliths having signal attenuation at higher values that would be consistent with calcification.

**TABLE 4.** Retrospective Radiology Review of Appendicoliths

	All Cases With Radiologic Appendicoliths (n = 42)	Cases With Only CT Seen Appendicoliths (n = 17)	Cases With CT and Path Seen Appendicoliths (n = 25)
Length of appendicolith in mm [min, mean, max (SD)]	2, 8.55, 21 (3.83)	2, 8.35, 21 (4.84)	3, 8.68, 14 (3.05)
Width of appendicolith in mm [min, mean, max (SD)]	2, 5.90, 12 (2.34)	2, 4.94, 10 (2.19)	2, 6.56, 12 (2.24)
Mean Hounsfield units of entire appendicolith [mean, (SD)]	299.1 (288.33)	217.4 (148.96)	354.6 (345.39)
Max Hounsfield unit of appendicolith [mean, (SD)]	388.0 (341.24)	255.2 (160.59)	478.28 (400.95)
Appear to be obstructing on CT	30 (71.4%)	12 (70.6%)	18 (72%)
Dilated appendix	40 (95.2%)	16 (94.1%)	24 (96%)
Surrounding inflammation	41 (97.6%)	17 (100%)	24 (96%)
Caliber change around appendicolith	29 (64.3)	13 (76.5%)	16 (64%)
Total appendix length (mm)	77.98 (25.90)	81 (27.39)	75.7 (25.13)
Base to appendicolith distance (mm; median, IQR)	26.5 (18, 44)	23.1 (15, 39.5)	29.0 (21, 39)
Percent length from base to first appendicolith	34.4% (34.68%)	31.6% (41.56%)	36.4% (29.42%)
Average number of appendicoliths	1.76 (1.14)	1.94 (1.30)	1.64 (1.04)
Appendicolith at tip or unlikely cause of appendicitis	5 (11.9%)	2 (11.8%)	3 (12%)

IQR, interquartile range.

**FIGURE 3.**
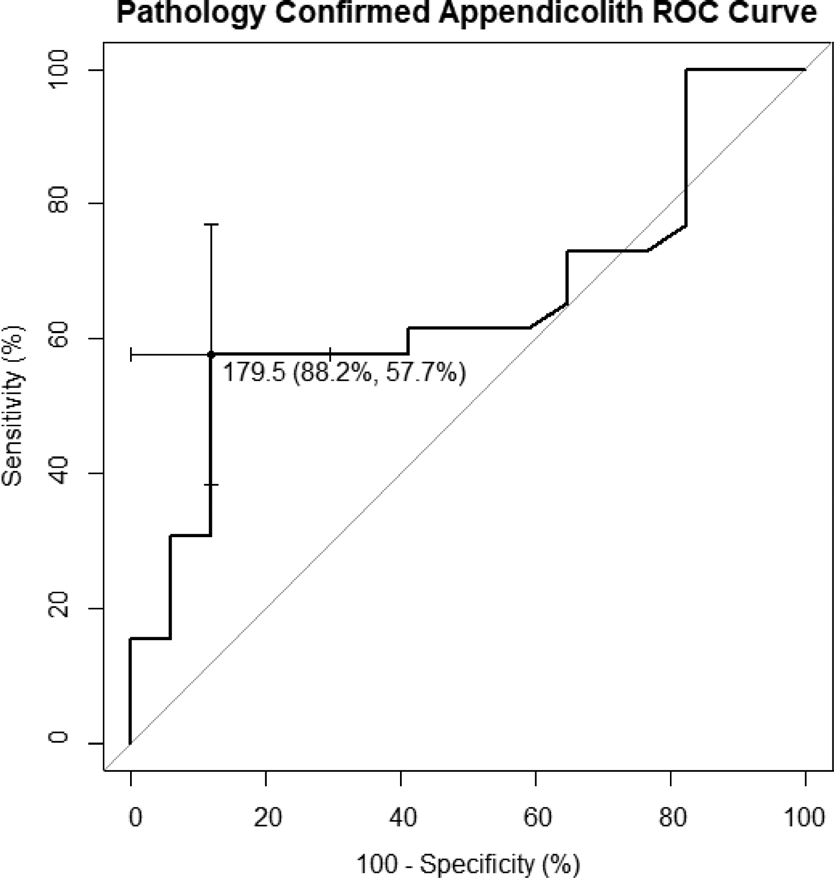
HU threshold analysis. The effect of varying the HU threshold difference between a luminal object and the maximal HU of the adjacent appendiceal wall. The upper, dashed line is the true-positive rate (sensitivity) and the lower, solid line is the false-positive rate (1-specificity). At 20 HU, the true-positive rate is 1, meaning that all appendicoliths that were identified at pathology would be identified on preoperative imaging. The optimal threshold is 180–200 HU that strikes the best balance between the true- and false-positive rates.

ROC analysis of 43 CT images associated with cases that had pathological confirmation of the presence of appendicolith found that the optimal threshold for having the greatest likelihood of encountering an appendicolith after appendectomy was 179.5 HU. HU refers to the difference in HU between the intraluminal mass thought to be an appendicolith and the maximal HU of the appendiceal wall. At this threshold, the sensitivity (true positive rate) for predicting a pathologically confirmed appendicolith when one is seen on preoperative CT imaging was 58%. The specificity for not finding an appendicolith and one not being there at pathology (1 – the false positive rate) was 88%. This translates to a LR of 4.9.^8^ The balance between differing true and false positive with varying HU thresholds is shown in Figure 3.

Of the 40 cases with both CT findings of dilated appendix and presence of appendicolith, 30 were thought to be obstructing and 10 nonobstructing based on the presence or absence of distal appendiceal caliber change. There were 7 appendicoliths found to be obstructing or partially obstructing the appendix on pathological examination. Of these, 6 were reported to have obstructing appendicoliths on CT and 1 did not have an appendicolith observed on CT. Appendicoliths were identified on CT preoperatively at the base of the appendix in 12 instances. Only 4 of the 12 (33.3%) were confirmed to be at this location when examined on pathology, compared with 17 of 48 (35.4%) overall.

After re-review of all preoperative imaging, 5 cases were identified with appendicoliths on CT in which the appendicolith was either at the tip of the appendix with inflammation much more proximal to the appendicolith, or with an apparent nonobstructing and free-floating appendicolith. These 5 cases of appendicitis were assessed to be unlikely due to the presence of appendicoliths based on the retrospective cross-sectional imaging review. Reasons for presuming appendicoliths were not obstructing were the location of appendicolith at the tip of the appendix with inflammation much more proximally, or small appendicolith appearing to float freely in the lumen with a noticeable size discrepancy between appendiceal lumen diameter and appendicolith diameter.

## DISCUSSION

The full clinical significance of appendicoliths remains unknown. Originally considered to be a strong risk factor for failure of NOTA,^2,3^ recent evidence suggests that they may predict failure, but to a lesser degree than previously thought.^4^ One problem with using a CT diagnosis of an appendicolith to guide NOTA is a lack of standardization for what is called an appendicolith and what it represents pathologically. In the current study, we found significant inconsistencies between imaging and pathological findings of appendicoliths. The most definitive, recent evidence relating preoperative CT-imaging findings of appendicoliths and NOTA treatment failure is from the Comparison of Outcomes of Antibiotic Drugs and Appendectomy (CODA) trial, where the presence of an appendicolith on preoperative CT was associated with 10% greater failure rate of NOTA. This low-failure rate might be explained by our observations. We found many cases where no appendicolith was seen in the resected appendix despite having a preoperative CT diagnosis of appendicolith. We also found very few appendicoliths examined pathologically that appeared to be clinically important. Thus, perhaps only relatively few appendicoliths identified on preoperative imaging are clinically significant.

Currently, there is no standard definition for what is labeled as an appendicolith on radiological or pathological examination of the appendix. One study examining the role of appendicoliths as a cause of appendicitis defined them as fecal concretions or pellets that may or may not have been calcified that were observed in the resected specimen.^9^ Appendicoliths were found in 18% of appendicitis cases and 29% of negative appendectomies, leading the authors to conclude that appendicoliths probably do not cause appendicitis. Another study examined appendicoliths found on preoperative CT defining them as high-density material in the appendix having similar attenuation to that of adjacent bone. This study did not correlate imaging findings with the pathology of the resected appendix.^10^ Another study involved a detailed analysis of the radiological appearance of appendicoliths but did not correlate the findings to pathological examination nor arrive at a definition for what should be called an appendicolith on CT imaging.^11^ Ranieri defined an appendicolith as a focal, calcific deposit within the appendiceal lumen finding them in 39% of cases when appendicitis was present compared with only 4% when there was no appendicitis.^12^ The variation in appendicolith incidence with appendicitis might be attributable to differing definitions of what an appendicolith is.

**FIGURE 4.**
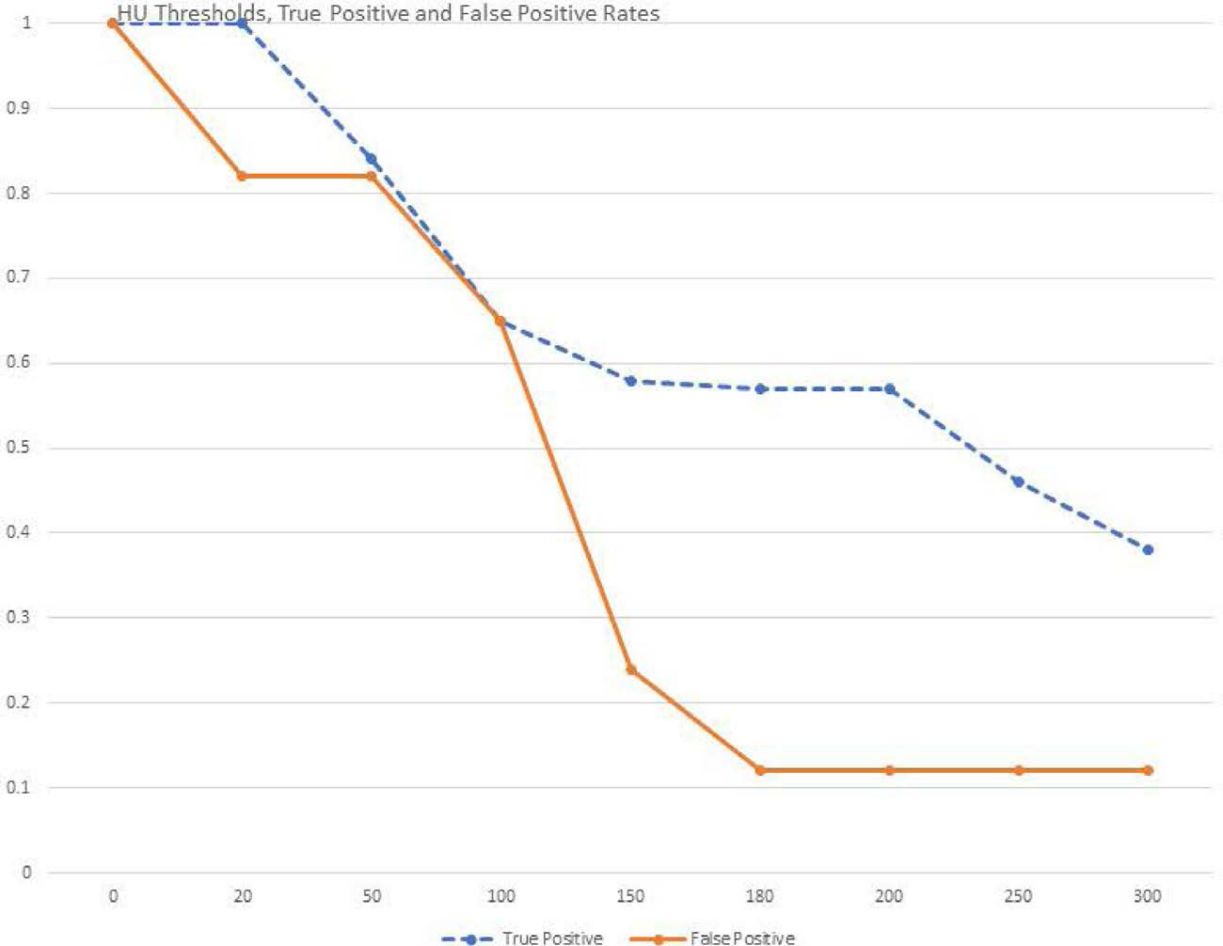
ROC for various CT-Hounsfield unit thresholds predicting pathologically present appendicoliths. ROC curves plot true positives on the *y*-axis vs false-positive rates (1-specificity) on the *x*-axis for various thresholds of a test—in this case, the difference in Hounsfield units between an intraluminal mass believed to be an appendicolith and the maximal Hounsfield unit measurement of the adjacent appendiceal wall. The optimal Housnfield unit cutoff is 179.5 yielding a sensitivity of 58% and specificity of 88.2%.

ROC analysis of the difference in maximal HU between the luminal structure thought to be an appendicolith and the appendiceal wall reveal a LR ratio of 4.9 for improving the diagnostic accuracy of appendicoliths found on CT for predicting their presence at pathology (Fig. 4). A LR of 4.9 means that there is a 4.9-fold higher probability of finding an appendicolith on CT if one is truly present than if an appendicolith is not present at the time of pathology.^5^ The prevalence, and thus the pretest probability, of appendicolith in acute appendicitis in our study was 32% (28 pathologically confirmed appendicoliths of 88 cases). If an appendicolith, as defined as having a greater HU density than the appendiceal wall by at least 180 HU, is found on CT, the probability of an appendicolith being found at pathology increases from the expected rate of 32% based on the prevalence of appendicolith to 70%.^8^

Appendicoliths are concretions of feces that may or may not be calcified. The relatively high false-negative appendicolith rate for using a HU threshold of 180 suggests that some appendicoliths are calcified and others are not. How this relates to the pathological importance of these lesions as contributors to failed NOTA is not known.

**FIGURE 5.**
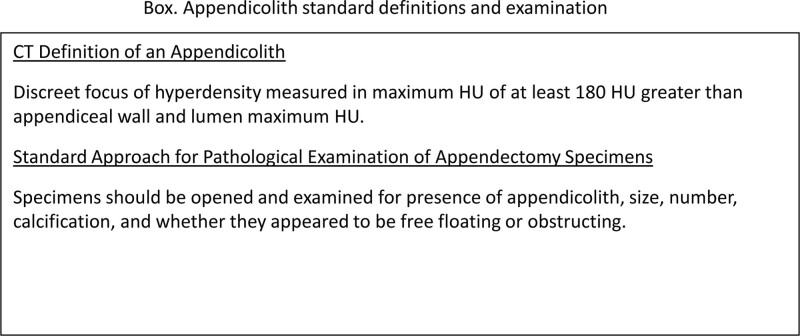
Proposed new definitions for what to call an appendicolith on CT scan. The proposed HU threshold maximizes the sensitivity and specificity for CT diagnosis of appendicolith. Also proposed is a standard approach to pathological examination of the resected appendix.

Going forward, we propose a standard definition for what is called an appendicolith on CT and a standard approach for pathological examination of the resected appendix. Standardization will improve data collection in the future, facilitating better understanding of the pathophysiology of appendicitis and the role of appendicoliths in the disease. For CT imaging, we propose diagnosing the presence of an appendicolith when there is a discreet hyperdense mass having a maximum HU of at least 180 HU greater than appendiceal wall and lumen maximum HU (Fig. 5). To collect data that will improve the prognostic significance of CT findings of appendicolith, the difference between the luminal mass HU and maximal HU for the appendiceal wall should also be reported. Resected specimens should be opened and examined for the presence of appendicolith, size, number, calcification, and whether they appeared to be free-floating or obstructing.

Appendicitis might be caused by luminal obstruction, behaving as if there was a bowel obstruction of the appendix. This possibility was investigated in detail by Wagensteen, who performed a series of animal and human investigations of appendicitis.^13,14^ In animal experiments, both increased intraluminal pressure and a bacterial inoculum were required to cause a clinical condition mimicking human appendicitis. Neither infection nor pressure alone were sufficient to cause appendicitis. In human studies, Wagensteen found appendicoliths in only 44% of appendicitis cases. Given the finding that a minority of appendicitis cases were associated with appendicoliths, Wagensteen posited that lymphoid hyperplasia or stricture of the appendiceal base resulted in obstruction and increased intraluminal pressure necessary to cause appendicitis. Wagensteen measured appendiceal intraluminal pressures while performing human appendectomy, finding them to be elevated. These observations led to the conclusion that there was an entity he called appendicular colic, a condition causing abdominal pain caused by obstruction and distention of the appendix that is not associated with infection and might explain negative appendectomies.

A novel aspect of our study is that the pathological examination of resected specimens followed a protocol established at its outset to standardize findings. There was no protocol defining what is called an appendicolith on preoperative CT imaging, which may have led to some of the discordance between radiological and pathology findings in our study. In developing our radiology re-read protocol, it was noted that because of their uncertain clinical significance, radiologists did not always mention appendicoliths in CT reports. When we began this study, our intent was to determine characteristic findings on CT for clinically important appendicoliths. However, only two thirds of appendicoliths seemed to be clearly obstructing the appendiceal lumen. This suggests that either the mechanism by which appendicoliths are clinically significant is not solely via obstruction, and that many appendicoliths are not clinically significant in appendicitis.

Our study examined all appendicoliths identified radiographically at our institution in adults. In this population, CT is the imaging modality of choice for patients presenting emergently with acute abdominal pain. In this series, only 2 patients underwent ultrasound imaging. However, in the pediatric population, ultrasound is the imaging modality of choice given risks of radiation and paucity of intraabdominal adiposity. However, ultrasound is being increasingly used for the identification of appendicitis in the adult population as well. Appendicoliths are visualized with ultrasound as hyperechoic lesions with associated posterior shadowing.^15^ Prior studies examining the relationship between the findings of appendicolith on ultrasound and appendicitis have yielded equivocal results. One study did report an association (odds ratio, 15.7; 95% CI, 1.42–174.6) but the extremely wide confidence intervals call into question the reliability of this finding.^16^ There is a need to better understand the relationship between appendicoliths observed on ultrasound and appendicitis.

## CONCLUSIONS

Discrepancies were observed between CT and pathology findings of appendicoliths. Not all appendicoliths seem to cause appendicitis. Because the presence of appendicolith influences the treatment decisions, there is a need to standardize their radiological diagnosis and better understand their pathophysiology.

## ACKNOWLEDGMENTS

Z.N.W. participated in research design, writing of the article, performance of the research, and data analysis. A.C. and B.V.N. participated in research design, writing of the article, and performance of the research. D.G. participated in research design and writing of the article. E.H.L. participated in research design, writing of the article, and data analysis.
